# Atypical femoral fracture mimicking lumbar radiculopathy in two patients taking bisphosphonate long-term: A case report

**DOI:** 10.1186/s12891-022-05990-7

**Published:** 2022-12-17

**Authors:** Koki Tsuchiya, Ichiro Okano, Katsuyuki Shiose, Yoshifumi Kudo, Chikara Hayakawa, Takuma Kuroda, Tomoaki Toyone, Katsunori Inagaki

**Affiliations:** 1 Department of Orthopaedic Surgery, Showa University School of Medicine, 1-5-8 Hatanodai, Shinagawa, Tokyo, 142-8666 Japan; 2 Department of Orthopaedic Surgery, Yamanashi Red Cross Hospital, 6663-1 Funatsu, Fujikawaguchiko, Yamanashi, 401-0301 Japan

**Keywords:** Lumbar spinal canal stenosis, Atypical femoral fracture, Osteoporosis

## Abstract

**Background:**

Atypical femoral fracture (AFF) is a rare complication in patients with osteoporosis undergoing long-term bisphosphonate therapy. The most common symptom of incomplete AFF is nonspecific thigh pain, which is often difficult to distinguish from other causes.

**Case presentation:**

We present two cases of AFF misdiagnosed as lumbar radiculopathy. Both patients visited our hospital for thigh pain, and in both cases the findings of lumbar spine magnetic resonance imaging showed substantial nerve compression. These patients had been treated for lumbar radiculopathy, but localized symptoms became conspicuous and femoral radiographs revealed complete AFF. The initial radiographs were reviewed retrospectively and revealed slight lateral cortical thickening in the affected femur, denoting a missed incomplete AFF. Internal fixation with intramedullary nails was performed.

**Conclusions:**

AFF may mimic lumbar radiculopathy. Therefore, clinicians should consider AFF as a differential diagnosis in older patients with lumbar canal stenosis who are undergoing long-term bisphosphonate therapy and present with thigh pain.

## Background

Bisphosphonates are the most commonly prescribed medications for osteoporosis that prevent osteoporotic fractures [1]. Several complications have been reported with bisphosphonate therapy, with atypical femoral fracture (AFF) classed as one of the most challenging conditions. AFF is principally observed in patients undergoing long-term bisphosphonate treatment [2].

The mean age of patients with surgically-treated, bisphosphonate-related AFF is reportedly 70 years, with groin and thigh pain being the most common symptoms [3]. Further, one study reported that 70% of patients with AFF had a history of prodromal pain in these regions [4]. The differential diagnoses of leg pain include lumbar lesions; moreover, the prevalence of lumbar pathology is high among older patients. Previous studies have shown that 90–100% of patients aged 65 years or over have disc degeneration, facet joint osteoarthritis, and/or osteophytes visible on lumbar spine radiographs [5, 6].

Therefore, the epidemiologic features and symptoms of AFF and lumbar radiculopathy are similar and these two conditions may coexist. However, missed incomplete or nondisplaced AFFs are at high risk of further displacement and may negatively impact the hospital stay, systemic or local complications, and/or mortality [7]. Thus, an accurate diagnosis is vital for the management of incomplete or nondisplaced AFF. Herein, we report two cases of missed AFF with lumbar lesions found on diagnostic imaging.

## Case presentations

### Case 1

A 67-year-old woman visited our hospital with a chief concern of left anterior thigh pain for 1 month without any specific history of trauma. The patient had a history of systemic lupus erythematosus (SLE) and osteoporosis, for which she had been administered a proton pump inhibitor (PPI), 10 mg prednisolone, and monthly alendronate for 5 years.

The patient had long-term sensory disturbance in the lower extremities owing to the peripheral neuropathy associated with SLE. The patient reported no rest pain in the left lower extremity, but reported pain during weight-bearing and walking. Further, mild weakness of the iliopsoas and quadriceps muscles was observed, probably because of pain and long-term disuse.

**Fig. 1:**
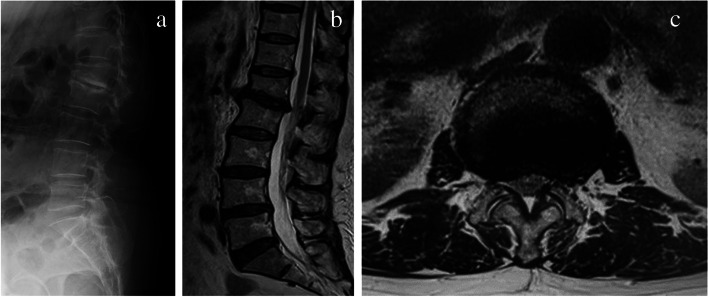
Radiological and MRI scan images of the lumbar spine in Case 1. **a** Radiological lateral image showing degenerative changes with loss of disc height at the level of L1-2. **b** Sagittal MRI showing lumbar canal stenosis (LCS) at L1-2. **c** Axial MRI showing LCS at L1-2

**Fig. 2:**
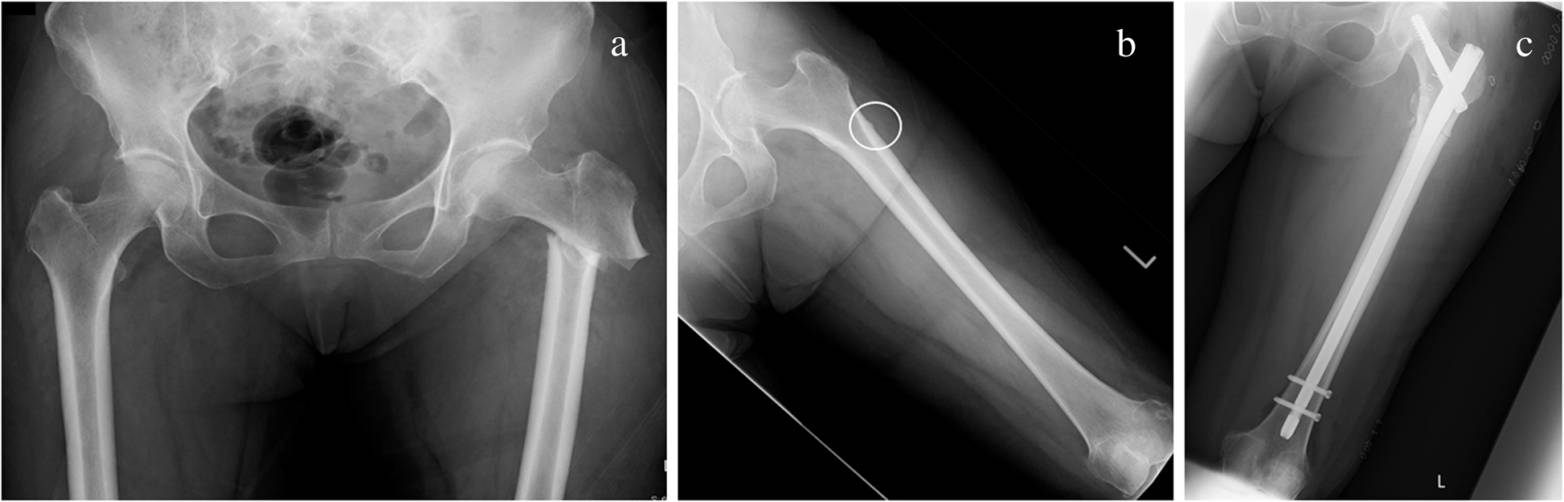
Femoral radiographs from Case 1. **a** The preoperative antero-posterior (AP) pelvis and hip view showing a subtrochanteric fracture of the left femur. **b** The AP femur view of the initial radiographs, showing lateral cortical thickening consistent with atypical femoral fracture (indicated by the white circle). **c** The postoperative AP view of the left femur showing the reduction of the fracture with an intramedullary nail

Metabolic studies and tests yielded normal results except for 25-dihydroxyvitamin D (serum calcium level, 9.1 mg/dl; serum phosphorus level, 4.1 mg/dL; and serum 25-dihydroxyvitamin D level, 14.4 ng/mL). Her initial radiographical examination included a lumbar spine series and antero-posterior (AP) pelvis and revealed degenerative changes with reduced disc height at L1-2 (Fig. 1a). Magnetic resonance imaging (MRI) of the lumbar spine showed degenerative disc disease, endplate changes, and lumbar canal stenosis (LCS) at L1-2 (Fig. 1b & c). The patient was diagnosed with LCS and posterior decompression surgery was scheduled. However, before the planned surgery she fell onto her hip. Radiographs of the left femur showed a left transverse femoral subtrochanteric fracture and characteristics of AFF (Fig. 2a). The initial radiographs were reviewed retrospectively and lateral cortical thickening of the proximal femur was noted (Fig. 2b). No obvious contralateral lesion was observed on the preoperative radiographs, and monthly alendronate administration was changed to daily teriparatide. The patient was treated surgically with intramedullary nails and the fracture healed without any complications (Fig. 2c).

### Case 2

**Fig. 3:**
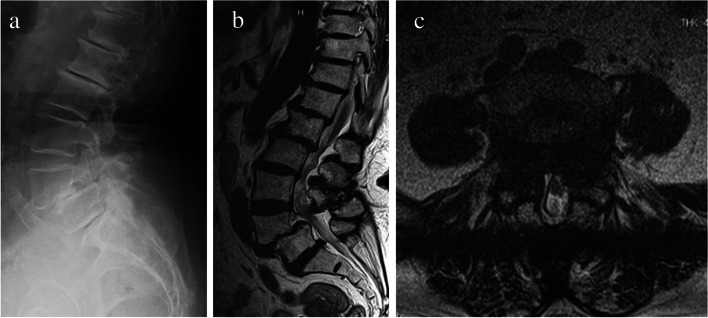
Radiological and MRI images of the lumbar spine in Case 2. **a** Radiological lateral lumbar image showing degenerative changes with loss of disc height at the level of L4-5 and grade 2 spondylolisthesis between L4 and L5. **b** Axial MRI image showing lumbar canal stenosis (LCS) at L4-5. **c** Sagittal MRI image showing LCS at L4-5

**Fig. 4:**
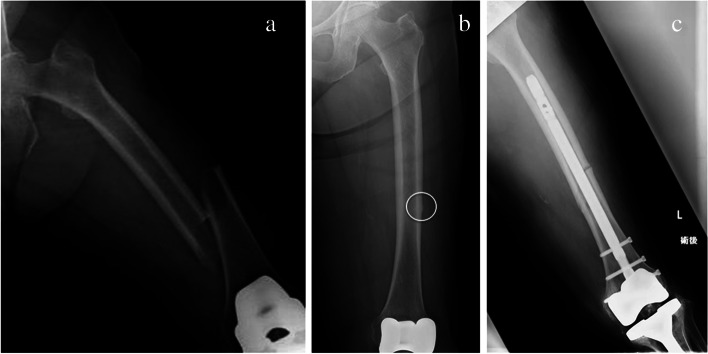
Femoral radiographs from Case 2. **a** The preoperative antero-posterior (AP) femur view showing a femoral shaft fracture on the left. **b** The AP femur view of the initial radiographs, showing lateral cortical thickening consistent with atypical femoral fracture (indicated by the white circle). **c** The postoperative AP view of the left femur showing the reduction of the fracture with an intramedullary nail

An 83-year-old woman with a history of breast cancer and osteoporosis visited our hospital with a chief concern of left lateral thigh pain for 2 weeks. She had been receiving PPI and monthly alendronate for 10 years for the management of osteoporosis. The patient had no obvious sensory disturbance or muscle weakness. She had no rest pain in the left lower extremity, but had pain during weight-bearing and walking*.* Metabolic studies and tests yielded normal results except for 25-dihydroxyvitamin D (serum calcium level, 10.2 mg/dl; serum phosphorus level, 3.4 mg/dL; and serum 25-dihydroxyvitamin D level, 18.8 ng/mL). The initial radiographical examination included a lumbar spine series and revealed decreased disc height and grade 2 spondylolisthesis at L4-5 (Fig. 3a). A lumbar spine MRI showed severe lumbar LCS at L4-L5, as well as narrowing of the bilateral foramina at L4-L5 (Fig. 3b & c). Selective nerve root blocks were performed on L4 and L5, which were partially effective. The following day, she had a fall and radiographs of the femur revealed a left transverse mid-shaft femoral diaphyseal fracture, showing features of AFF (Fig. 4a). The initial radiographs were reviewed retrospectively and lateral cortical thickening of the femur diaphysis was observed (Fig. 4b). No obvious contralateral lesion was observed on the preoperative radiographs, and monthly alendronate administration was changed to romosozumab. She underwent surgical repair with a retrograde intramedullary nail and the fracture healed (Fig. 4c).

## Discussion and conclusions

LCS is often difficult to diagnose as a large number of differentials can mimic this condition, including herpes zoster, peripheral arterial disease, peripheral neuropathy, [8, 9] and pathologies of the pelvis, hip joint, and femur [10]. In the study of Schneider et al., [11] the authors mentioned that lumbar MRI was used for the differential diagnosis probably in certain patients with AFF. However, detailed MRI findings and clinical symptoms related to the lumbar spine were not well described. This is of value since it reports the detailed clinical information which helps treating physicians to guide correct diagnosis and proper treatment.

AFF is a type of stress fracture called an insufficiency fracture that develops over time, most commonly after prolonged suppression of bone remodeling by the bisphosphonates [2]. Repetitive loading of the femur may cause micro-cracks which usually heal asymptomatically. In certain circumstances, such as with prolonged bisphosphonate treatment, these micro-cracks may accumulate in the cortex and eventually cause a complete fracture. The absolute incidence of AFF remains low. In a systematic review of 14 studies, [12] the incidence ranged from 3.0 to 9.8 cases per 100,000 patient-years. However, long-term bisphosphonates usage (> 5 years) increases the absolute risk of AFF to 113 cases per 100,000 patient-years. The first American Society of Bone and Mineral Research (ASBMR) task force revealed that 92% of AFF cases were treated with bisphosphonates for osteoporosis, and the mean duration of bisphosphonates therapy was 7 years [4]. Moreover, other risk factors for AFF must be considered; Asian women had an eight times higher risk than white women [13] and other drugs associated with AFF include glucocorticoids and PPI [14, 15]. Therefore, the present cases were considered to be at high-risk for AFF.

A systematic review found that the mean reported age range of patients with bisphosphonate-related AFF who had surgical treatment was 66.8–74.2 years [3]. In the 2010 ASBMR task force, dull or arching pain in the groin or thigh were noted as prodromal symptoms. However, lumbar pathologies are also major causes of thigh pain in older patients, and previous studies have reported symptomatic lumbar stenosis occurs in approximately 10% of the population over the age of 70 years. Moreover, asymptomatic MRI findings, such as lumbar disc bulge and herniation, are common. One study demonstrated that approximately 80% of patients aged over 70 years had asymptomatic lumbar lesions on MRI findings [16]. In the present two cases, the coexistence of AFF and radiological lumbar stenosis led to a delay in the diagnosis of AFF. Acute radiculopathy begins at the onset of symptoms and lasts up to 4–6 weeks [17]. On the other hand, the duration of AFF symptoms has been reported to be < 1–18 months [18]. Considering the symptoms of acute radiculopathy and AFF, it is difficult to differentiate between the two diseases based on symptom duration alone. Furthermore, previous studies have reported hip osteoarthritis and stress fracture as mimickers of lumbar radiculopathy [8, 10]. Especially, stress fractures of the femoral neck show insidious onset of groin pain that worsens with running or marching and improves with rest. These symptoms are similar to LCS symptoms. The two patients had weight-bearing groin and thigh pain; therefore, hip femur lesions should have been considered as differential diagnoses. Owing to the similar distribution of pain, distinguishing AFF from lumbar radiculopathy with physical examination alone may be difficult, and multimodal diagnostic work up including additional radiographic examinations should be warranted.

Although the presence of the dreaded black line on the initial radiographs is a radiologic feature indicative of stress fracture nonunion, [19] the dreaded black line was not observed in either patient.

Delayed or missed diagnosis of AFF, especially in the case of an incomplete fracture, may negatively impact the patient’s prognosis, as seen in the two cases presented where the fractures both progressed from incomplete to complete. Banffy et al. reported that five of six incomplete AFFs progressed to complete AFF, and patients who underwent surgery for incomplete AFF had shorter hospital stays than those of patients with complete AFF [7]. Careful history-taking with respect to the exact nature of the pain should be warranted for differentiating between AFF and lumbar radiculopathy, especially when lumbar stenosis is identified on MRI.

In summary, we reported two cases of AFF mimicking lumbar radiculopathy. Clinicians should consider AFF as a differential diagnosis in older patients with LCS who are undergoing long-term bisphosphonate therapy and present with thigh pain.

## Data Availability

Data that support the findings of this study were not deposited publicly due to the data sharing policy of Department of Orthopaedic Surgery, Showa University, but are available from the corresponding author on reasonable request.

## Abbreviations

AFF: Atypical femoral fracture
AP: Antero-posterior
LCS: Lumbar canal stenosis
MRI: Magnetic resonance imaging
PPI: Proton pump inhibitor
SLE: Systemic lupus erythematosus
